# An Aboriginal community-led approach to reducing alcohol-related harm: A multiple baseline, stepped wedge evaluation

**DOI:** 10.1016/j.puhip.2025.100689

**Published:** 2025-12-13

**Authors:** Mieke Snijder, Annemarie Wagemakers, Bianca Calabria, Bonita Byrne, Jamie O'Neill, Ronald Bamblett, Chiara Stone, Alice Munro, Christopher Oldmeadow, Simon Chiu, Anthony Shakeshaft

**Affiliations:** a Institute of Development Studies, Brighton, United Kingdom; b Health and Society, Wageningen University & Research, Wageningen, the Netherlands; c Centre for Mental Health Research, Research School of Population Health, Australian National University, Canberra, Australia; d National Drug and Alcohol Research Centre, UNSW Sydney, Australia; e Community 1, Australia; f Community 2, Australia; g Community 3, Australia; h Western New South Wales Local Health District, Orange, Australia; i Hunter Medical Research Institute, Newcastle, Australia; j Sydney University, Sydney, Australia; k Poche Centre for Indigenous Health, University of Queensland, Australia; l Discipline of Psychology, University of the Sunshine Coast, Queensland, Australia

**Keywords:** Aboriginal, Indigenous, Community, Health, Alcohol, Rural

## Abstract

**Objectives:**

Three Aboriginal communities in regional Australia led the development and implementation of a community-based program aimed at: i) reducing alcohol-related criminal incidents; and ii) improving community perceptions of community safety and empowerment.

**Study design:**

A multiple baseline, stepped-wedge evaluation.

**Methods:**

The co-designed program comprised community-specific activities to operationalise three core components that were standardised across all communities: i) improving service engagement; ii) promoting community activities; and iii) increasing community members’ empowerment for action. Outcome measures were de-identified crime data (persons of interest and victims from January 1, 2005 to December 31, 2017) and pre/post community surveys.

**Results:**

Statistically significant improvements in perceptions of alcohol harm were reported in all three communities: i) community 1 significantly increased community empowerment; ii) community 2 reported significantly less alcohol-related verbal abuse and injuries, and feeling significantly safer during the day and at night; and iii) community 3 reported feeling significantly safer at night. There were no statistically significant reductions in alcohol-related crime.

**Conclusion:**

This is the first Aboriginal-specific, community-based project in Australia to use a multiple baseline, stepped-wedge evaluation design and an innovative program logic model. Future research could seek to uncover the mechanisms associated with different program impacts on different outcomes and in different communities, and seek to sustain impacts over longer timeframes.

## 1 Introduction

The effects of colonisation continue to be experienced by Aboriginal and Torres Strait Islander people and communities in Australia, through intergenerational trauma and its related consequences. One of the most visible manifestations of this ongoing trauma is their disproportionately greater exposure to harm from alcohol and other drugs [1]. Despite recent progress in reducing the impact of these harms [2], an estimated 12 % and 8 % of the total burden of disease remain attributable to tobacco and alcohol use respectively, and together they account for 14 % of the gap in health status between Indigenous and non-Indigenous Australians [3]. Specifically for alcohol, harms appear to be predominantly related to excessive, single occasion drinking, given Indigenous Australians are less likely to drink any alcohol compared to non-Indigenous Australians [4].

Community-based responses are potentially well placed to reduce harms associated with alcohol and other drugs because they can augment close community relationships, foster positive role models and strengthen connection to culture [5]. There is emerging evidence that community-based programs can be effective in reducing alcohol-related harms in Indigenous communities [6]. Community-based participatory research (CBPR), for example, is one framework that utilises Indigenous self-determination and empowerment by involving communities as partners and leaders in every step of the research process [[7], [8], [9], [10]]. This type of meaningful community participation enhances the relevance of research to communities and builds on their existing strengths.

For this study, three Aboriginal communities in regional New South Wales (NSW), Australia, led the development and implementation of a community-based program using the principles of CBPR (note that the Aboriginal Health and Medical Research Council of NSW prefers to use the term ‘Aboriginal’ in recognition that Aboriginal peoples are the original inhabitants of NSW [11]). The specific aims were to: i) reduce alcohol-related criminal incidents; and ii) improve community perceptions of community safety and empowerment.

## 2 Methods

### 2.1 Evaluation design

**Fig. 1:**
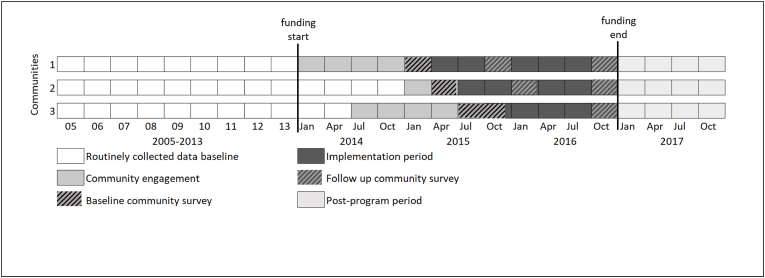
Overview of the evaluation design.

As delineated in Fig. 1, a multiple baseline, stepped-wedge evaluation design was used, in which each community commenced the program in different months: 12th April (community 1); 7th July (community 2); and the 19th December (community 3) 2015 [12]. The logic of this design is that data are analysed and reported separately for each community because each community acts as its own control (the multiple baseline component), while the staggered introduction of the program into each community at different points in time (the stepped-wedge component) optimises the likelihood that any post-program changes are due to the program, rather than some other co-occurring variable [12]. It was not possible to randomise the order in which the program commenced in each community due to the unique requirements for community-specific engagement. The multiple baseline component of this design is the nine-year baseline period (2005–2013), established using retrospective, routinely collected crime data from each community.

### 2.2 Participating communities and project members

The three participating communities were selected because the largest Aboriginal Community Controlled Health Organisation (ACCHO) in the region advised that reducing alcohol harm in these communities was a priority. Communities 1 and 3 were regional communities with populations of 24,000 (4.1 % Aboriginal) and 5700 (10.3 % Aboriginal), respectively, and community 2 was a remote community with a population of 1250 (26.4 % Aboriginal) people [13]. Both Aboriginal and non-Aboriginal individuals from these communities were invited to participate in any aspect of the development, implementation or evaluation of the program to promote inclusivity and build community cohesion. Community members were invited to participate through four mechanisms: i) advertisements in local newspapers, radio programs and school newsletters; ii) word-of-mouth; iii) local health services encouraging their patients to participate; and iv) liaising with local youth services to engage high-risk young people.

### 2.3 Community-based program

**Fig. 2:**
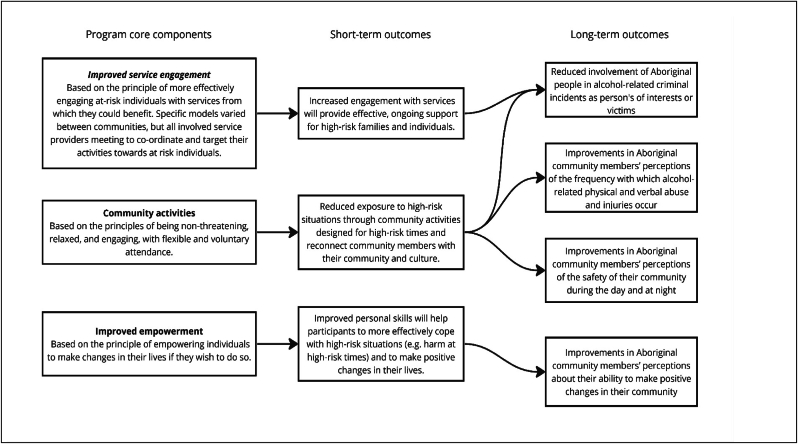
The three core components of the community-based program, aligned with their anticipated short- and long-term outcomes.

A detailed description of the development of the community-based program has been provided elsewhere [14]. Note that the term ‘program’ is used throughout this paper, rather than ‘intervention’, due to the negative and paternalistic connotations of the Northern Territory's Emergency Response (or ‘the intervention’) that was imposed by the Australian Government on the Indigenous people of the Northern Territory between 2007 and 2012. A project-specific Implementation Committee was established in each community to design the program. This committee comprised local Aboriginal community members, Aboriginal and non-Aboriginal service providers, the Aboriginal project co-ordinator in each community (authors BB, JO and RB) and members of the research team (primarily authors MS and AS). As shown in Fig. 2, a highly innovative aspect of the community-based program was to standardise it across all three communities by defining it as comprising three core components, and then aligning those core components with short-term outcomes (based on the rationale for how each core component should work) and long-term outcomes (the measurable impact of the program). The three core components were: i) improving service engagement; ii) promoting community activities; and iii) increasing community members' sense of empowerment for action.

The three core components identified in Fig. 2 were actioned through the development and implementation of the activities summarised in Table 1. These activities, and the mechanism for their implementation, were developed by the Implementation Committee in each community, whose members selected a suite of activities that reflected the strengths and interests of their own individual communities. Where possible, these activities were integrated into existing services to promote their sustainability, such as local youth or health organisations. Where these services did not exist, the selected activities were implemented by the Aboriginal project coordinators. Each Implementation Committee was provided with a detailed description of the project budget, which they agreed to allocate equally in each community to employ the Aboriginal project co-ordinator and to provide AUD$50,000 for the implementation of their selected activities. Table 1 summarises the activities in each core component, the number of sessions of each activity that were provided and the total number of people who engaged in each activity.

**Table 1:** The specific activities implemented in each community, including the number of sessions and total number of engagements with each activity.

Core Components	Community 1 (commenced 20 April 2015)				Community 2 (commenced 7 July 2015)				Community 3 (commenced 19 December 2015)		
	Activities	No. sessions	Total engagements^a^		Activities	No. sessions	Total engagements^a^		Activities	No. sessions	Total engagements^a^
1. Improved service engagement	Case coordination	5	15		Case coordination	2	20		Engagement Officers	n/a	2
	Skills training	36	180		Engagement Officers	n/a	6				
	Engagement Officers	n/a	4								
2. Community activities	Midnight Basketball 1	8	293		Touch football	7	180		Movie	2	26
	Midnight Basketball 2	8	319		Outdoor movie	1	97		Country music	2	26
	Midnight Basketball 3	8	352		Cultural activities	4	222		Community meeting	2	28
	Midnight Basketball 4	8	344		Boxing classes	19	228		Cultural activities	5	80
	Cultural activities	3	45		Holiday program	8	48		Night Basketball	6	113
	Billiards competition	4	60		Music night	1	85		Hip-hop dancing	6	36
	Skate Slam	1	26		Youth jam sessions	2	13		Indoor soccer	3	66
	Cultural sports day	1	397		Indoor basketball	4	131		Talent quest	3	75
	BBQ in the park	3	116		Beauty classes	3	16		Outdoor movie	2	500
	Family Bingo	3	163						Touch football day	1	25
	Movie night	2	56								
	Fitness Beyond Barriers	6	113								
	At-risk boys group	12	120								
	At-risk girls group	7	56								
3. Improved empowerment	Family Wellbeing	3	18		Family Wellbeing	5	50		Family Wellbeing	5	30
	Family Wellbeing	12	72						Self-esteem program	1	7
	Men's group FWB	4	12								
	School group FWB	6	60								
**Totals**	**21**	**140**	**2821**		**12**	**56**	**1096**		**13**	**36**	**1014**

a Given the community-level focus of the study (rather than an activity-level focus), only the total number of people who engaged in each activity is reported here, rather than a count of unique participants.

### 2.4 Measures

After the Implementation Committees determined their preferred activities to operationalise the three core components of the community-action program, the researchers devised a series of measures that could be used to assess the long-term outcomes of their programs. These measures were refined and finalised in consultation with the Implementation Committees to optimise their face-validity. As indicated in Fig. 2, these measures assessed both the involvement of Aboriginal people in harms reported to community-based services (routinely collected data) and community perceptions based on their lived experience of alcohol-related harm (community survey data).

#### 2.4.1 Routinely collected crime data

De-identified unit-record data for persons of interest (POI) and victims of criminal incidents were obtained from the NSW Bureau of Crime Statistics and Research for each community, for people aged 13 years and older and for the time period January 1, 2005 to December 31, 2017 [15]. As summarised in Fig. 1, these data were categorised into three study periods: i) baseline (January 1, 2005 to the program start date in each community); ii) implementation (program start date to December 31, 2016); and iii) post-program (January 1, 2017 to December 31, 2017). A POI is a suspected offender recorded by police in connection with a criminal incident, which may or may not be followed-up through the legal system. Since the involvement of alcohol is not recorded routinely, alcohol-related incidents were identified using an existing proxy measure [[16], [17], [18], [19]]. This proxy measure comprises offence types strongly associated with alcohol misuse (assault [domestic, non-domestic and police assault], sexual assault [sexual and indecent assault], malicious damage to property, and disorderly conduct [offensive conduct and offensive language]) that occur at times that are strongly associated with alcohol misuse (Thursdays, Fridays and Saturdays from 6pm to 6am). Only criminal incidents where the Aboriginal status of the victim or POI was known were included. The total number of Aboriginal POIs and victims of alcohol-related criminal incidents were aggregated into annual quarters.

#### 2.4.2 Pre- and post-program implementation community surveys

A 12-item survey was co-designed by the Implementation Committees, with items grouped into four domains. First, respondents were asked about their demographics: i) family group (their mob); ii) home country [20]; iii) age; iv) gender; v) education; and vi) employment status. Second, respondents were asked to rate their perceptions of alcohol harm using a scale from 1 to 10 (where 1 is never and 10 is very often) to indicate how often in their community: vii) a person who is affected by alcohol verbally abuses someone (which means, abusing with words, for example, saying mean things, swearing, threatening with words); viii) a person who is affected by alcohol physically abuses someone (for example, hitting or pushing someone); and ix) a person is injured or has an accident as a result of drinking alcohol) [21]. Third, respondents were asked to rate their perceptions of community safety using a scale from 1 to 10 (where 1 is very unsafe and 10 is very safe) to rate their perceptions of: x) the level of safety in their community during the day; and xi) the level of safety in their community during the night [21]. Fourth, based on the Indigenous-specific Growth and Empowerment Measure, respondents were asked to rate their perception of community empowerment using a scale from 1 to 10 (where 1 is completely unable and 10 is completely able): xii) how able their community is to make changes and improvements [22].

Aboriginal people aged at least 15 years residing in one of the three participating communities were eligible to complete the survey, irrespective of whether they had participated in a program activity. Participants comprised a convenience sample, stratified by gender and age (15–29 years or 30 years and older) [13]. A required survey sample size of 199 respondents across all three communities was estimated based on Cohen's d, using 80 % power, a conservative estimate of dz = 0.2 and α = 0.05 (two-sided test) [23]. This method was used because of a lack of previous trials that have measured alcohol-related injuries and violence in Aboriginal communities [24]. This estimated sample size was increased to 249 to allow for a 20 % loss to follow-up. Survey data were collected by local Aboriginal project coordinators, Aboriginal community researchers and non-Aboriginal members of the research team, approximately two months prior to program commencement through household door-knocking (65 %), at community events (6 %) and in the waiting room of the local ACCHO (29 %). Respondents were followed-up twice: 10–14 months after the baseline survey (all communities); and 24 or 18 months after baseline (communities 1 and 2, respectively). A second follow-up for community 3 was unable to be completed within the project funding timeline.

### 2.5 Statistical methods

Separately for each community, the effectiveness of the program was assessed as the impact on police incidents (administrative data) and lived experience (survey data) of the core, standardised program components, rather than the community-specific activities used to operationalise them. Specifically, police incidents involving Aboriginal POIs and victims were analysed using interrupted time series analyses, as is recommended for multiple baseline trials [25]. Generalised linear models for time series counts were used to specify segmented regression. Estimations were made using the conditional maximum likelihood for the negative binomial distribution. These models were implemented using the R package tscount [26]. Seasonality effects were addressed by adjusting for monthly and yearly lags separately, and by testing a factor variable (with levels for Autumn, Winter, Summer, Spring) using Cosine and Sine cyclical relationships. The time series segments allowed for shocks and changes in trends, and shifts in levels, caused by exogenous events. The program commencement dates were the main event of interest.

For the survey data, generalised linear mixed models for repeated measures were used to compare the first and second follow-up responses to baseline within each community, to assess the impact of the program on community perceptions of alcohol-related verbal abuse, physical abuse, injuries, community safety during the day, community safety at night and the capacity of the community to achieve change (empowerment). The analysis of survey data was conducted in SPSS 25 using 5 % significance levels.

## 3 Results

### 3.1 Program impact on crime

**Fig. 3:**
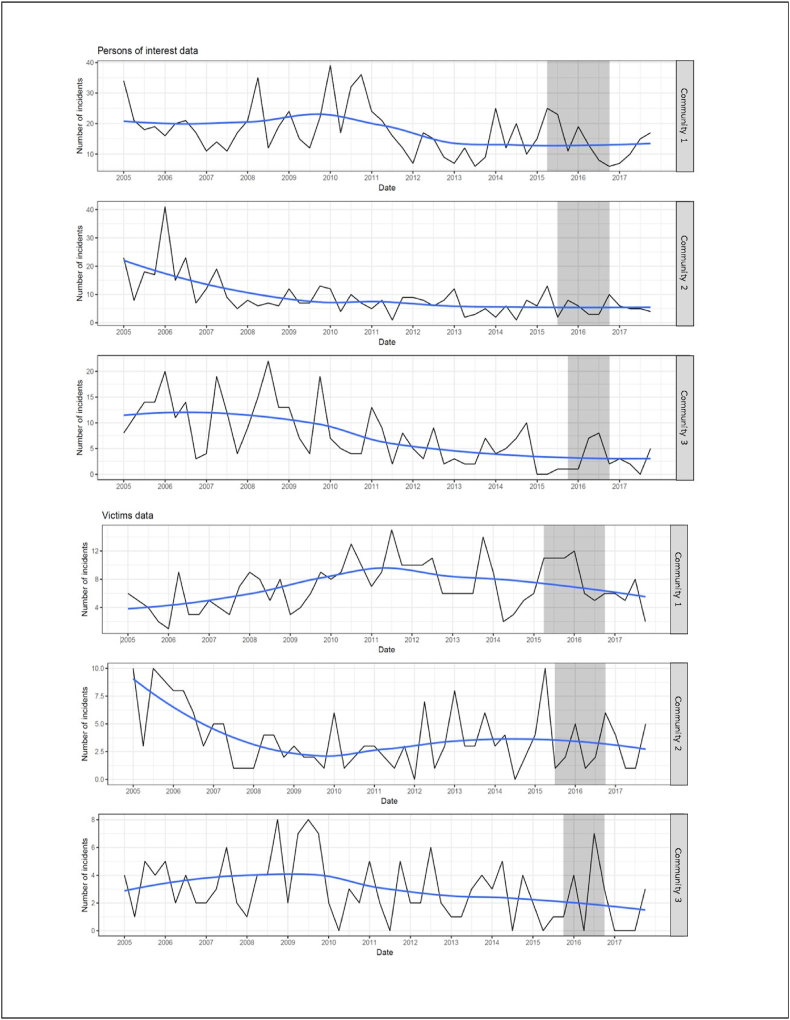
Quarterly trends in Aboriginal persons of interest and victims of alcohol-related crime.

Fig. 3 shows that in all communities there was a general downward trend in Aboriginal POI and victims of alcohol-related crime for the total study period (January 1, 2005 to December 31, 2017), except for victims of alcohol crime in community 1, which peaked about mid-2011. Table 2 shows that these downward trends were only statistically significant in the baseline period (January 1, 2005 to the commencement of the program) for POI in community 2 (IRR = 0.965, 95 %CI = 0.952–0.979, p < 0.001) and community 3 (IRR = 0.967, 95 %CI = 0.952–0.982, p < 0.001).

**Table 2:** Outcomes of generalised linear models for time series counts of Aboriginal persons of interest and victims in all three communities.

Outcome	Persons of Interest					Victims				
	IRR	Std Err	LCL	UCL	p-value	IRR	Std Err	LCL	UCL	p-value
Community 1										
Baseline trend	0.992	0.006	0.981	1.003	0.1631	1.009	0.006	0.996	1.021	0.1716
Program commencement shift	1.902	0.399	0.870	4.157	0.1072	1.676	0.372	0.809	3.475	0.1647
Program period trend	0.845	0.101	0.692	1.030	0.0953	0.852	0.852	0.704	0.704	0.0998
Post-program period shift	0.606	0.628	0.177	2.077	0.4258	1.520	0.581	0.487	4.745	0.4709
Post-program period trend	1.499	0.181	1.051	2.138	**0.0254∗**	1.014	0.180	0.180	1.442	0.9381

**Community 2**										
Baseline trend	0.965	0.007	0.952	0.979	**<0.001∗∗∗**	0.984	0.010	0.966	1.002	1.002
Program commencement shift	1.244	0.692	0.320	4.830	0.7523	0.704	0.948	0.110	4.520	0.7117
Program period trend	0.967	0.205	0.647	1.447	0.8717	1.064	0.273	0.623	1.817	0.8207
Post-program period shift	2.906	0.797	0.609	13.871	0.1810	2.159	1.017	0.294	15.851	0.4491
Post-program period trend	0.858	0.283	0.492	1.496	0.5895	0.824	0.367	0.402	1.690	0.5968

**Community 3**										
Baseline trend	0.967	0.008	0.952	0.982	**<0.001∗∗∗**	0.986	0.009	0.968	1.004	0.1232
Program commencement shift	0.141	1.185	0.014	1.434	0.0978	0.344	1.088	0.041	2.902	0.3266
Program period trend	2.137	0.373	1.028	4.443	**0.0419∗**	1.637	0.354	0.819	3.273	0.1633
Post-program period shift	0.175	1.041	0.023	1.343	0.0936	0.222	1.189	0.022	2.284	0.2057
Post-program period trend	0.549	0.454	0.225	1.337	0.1866	0.605	0.462	0.245	1.498	0.2776

IRR=Incidence Risk Ratio.

Std Err = Standard Error.

LCL = Lower Confidence Level.

UCL=Upper Confidence Level.

The program impact differs between communities. In community 1, the program period trends suggest fewer alcohol-related POI and victims while the program was being implemented, followed by increases in POI and victims after the program ceased, although the only statistically significant trend was for the increase in POI after the program ceased, as indicated by the POI post-program period trend (IRR = 1.499, 95 %CI = 1.181–1.051, p = 0.0254). In community 2, the program period trends and the post-program period trends suggest fewer alcohol-related POIs and victims during and after program implementation, except for victims during the program period. Nevertheless, neither of these trends were statistically significant. In community 3, the program period trends showed a statistically significant increase for POI while the program was being implemented (IRR = 2.137, 95 %CI: 1.028, 4.443, p = 0.0419). Although the post-program period trends suggest fewer alcohol-related POI and victims, neither trend was statistically significant.

### 3.2 Program impact on community perceptions

Across the three communities, a total of 239 surveys were completed. The demographic characteristics of the community survey participants are summarised in Table 3. Most respondents were females in all communities (range 55 %–69 %) and the median age of respondents was lowest in community 1.

**Table 3:** Demographic characteristics of the community survey participants.

Characteristics	Community 1				Community 2				Community 3	
	Baseline^e^	FU 1^a^	FU 2^b^		Baseline^e^	FU 1^a^	FU 2^c^		Baseline^e^	FU
Total participants (N = 239)	134	90 **(67 %)**^d^	73 **(54 %)**^d^		53	38 **(72 %)**^d^	29 **(55 %)**^d^		52	30 **(58 %)**^d^
Gender (Female)	82 (61 %)	50 (56 %)	40 (55 %)		35 (66 %)	22 (58 %)	20 (69 %)		32 (62 %)	18 (60 %)
Age					59					
Minimum	15	15	16		17	18	19		15	18
Maximum	71	72	72		79	73	73		78	70
Mean (median)	35 (32)	36 (32)	36 (33)		44 (47)	46 (50)	44 (50)		42 (43)	47 (49)
Unemployed	50 (37 %)	26 (29 %)	28 (38 %)		39 (74 %)	24 (63 %)	18 (67 %)		21 (43 %)	12 (40 %)
Education										
≤ Year 10 high school (about age 15/16 years)	75 (56 %)	53 (59 %)	42 (58 %)		39 (74 %)	27 (71 %)	22 (76 %)		33 (63 %)	18 (60 %)
≥ year 11 high school (≥ about age 17/18 years)	56 (42 %)	34 (38 %)	30 (41 %)		10 (19 %)	11 (29 %)	7 (24 %)		16 (31 %)	6 (40 %)

FU = Follow Up.

a FU 1 was 10–14 months after baseline in each community.

b FU 2 was 24 months after baseline in Community 1.

c FU 2 was 18 months after baseline in Community 2.

d Total participant percentages provided in bold text reflect the response (or attrition) rates for survey responses; other percentages reflect the proportion of respondents.

e Baseline surveys were conducted approximately 2 months before commencement of the program in each community.

**Fig. 4:**
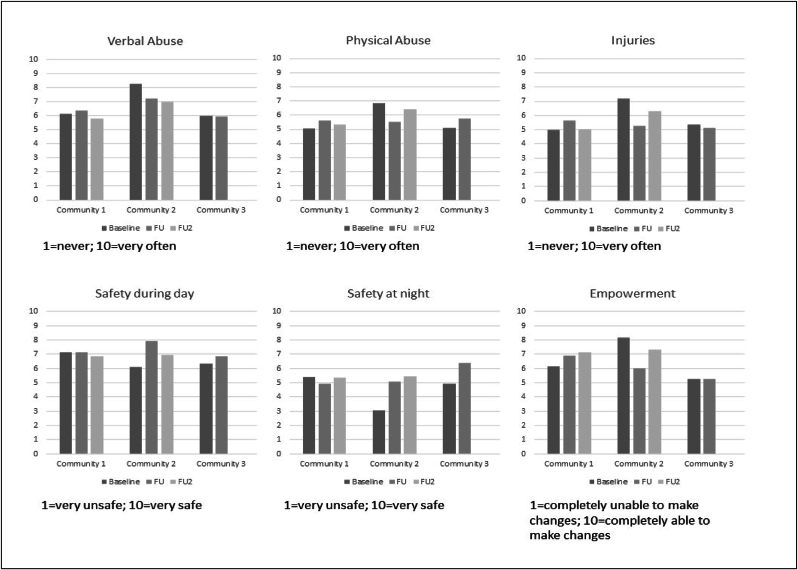
Findings from the community survey in each community.

Fig. 4 shows community perceptions of alcohol-related verbal abuse, physical abuse and injuries generally remained stable or declined between baseline and the two follow-up periods in all communities. In community 2, these observed reductions were statistically significant for alcohol-related injuries at the first follow-up (M = 5.25, SD = 2.98, *F*(2, 14) = 16.029, *p* = 0.001), and approached significance at the second follow up (M = 6.31, SD = 2.67, F(2, 14) = 3.540, p = 0.079), compared to baseline (M = 7.19, SD = 2.83).

Similarly, Fig. 4 shows community perceptions of feeling safer during the day, feeling safer at night and being empowered to achieve change generally remained stable or improved between baseline and the two follow-up periods in all communities. The one exception was in community 2, where the rating of community empowerment was statistically significantly lower at the first follow-up (M = 6.03, SD = 2.90, *F*(2, 14) = 4,621, *p* = 0.048) compared to baseline (M = 8.16, SD = 2.42), although this reduction was no longer statistically significant at the second follow-up (M = 7.31, SD = 3.27, *F*(2, 14) = 0.050, *p* = 0.826).

In community 1, community empowerment was not statistically significantly improved at the first follow-up (M = 6.89, SD = 2.13, *F*(1, 64) = 2.851, *p* = 0.096) but was statistically significantly improved at the second follow-up (M = 7.14, SD = 2.27, *F*(1, 64) = 8.787, *p* = 0.004), compared to baseline (M = 6.15, SD = 2.44). In community 2, perceptions of safety during the day statistically significantly improved at the first follow-up (M = 7.95, SD = 2.36, *F*(2, 14) = 16.029, *p* = 0.001), compared to baseline (M = 6.08, SD = 3.30). In community 3, perceptions of night-time safety statistically significantly improved at the first follow-up (M = 6.38, SD = 2.48, *F*(1, 11) = 9.801, *p* = 0.010), compared to baseline (M = 4.93, SD = 2.22).

## 4 Discussion

The survey data showed that the impact of the community-based program on participants’ lived experience was mixed. Despite alcohol-related verbal abuse, physical abuse and injuries generally remaining stable or falling between baseline and follow-up in all communities, these trends were only statistically significant in community 2 for injuries (at the first follow-up). Feeling safer during the day was significantly greater in community 2 (first follow-up). Feeling safer at night was significantly greater in community 3 (first follow-up). Community empowerment was significantly improved in community 1 at the second follow-up.

For the crime data, the only statistically significant findings were an increase in POI in community 3 and an increase in POI after the program ceased in community 1. The community 3 finding may be because their program commencement coincided with the start of the Christmas and summer holiday period. Greater flexibility in the timeframe for this project would have allowed postponing the commencement of the program to avoid this concurrence. Although it is possible that the cessation of the program in community 1 is causally related to the observed increase in POI post-program, this finding was not replicated for victims in community 1, nor in communities 2 and 3.

As for previous (Aboriginal and non-Aboriginal) community-based trials on alcohol harms, these findings demonstrate the difficulty of interpreting outcomes that derive from both routinely collected and self-reported data [6,19,27]. The greater number of statistically significant findings for the survey data may mean that community action approaches are more likely to improve the lived experience of the majority of community members (as reflected in their self-reported outcomes), rather than the routinely collected administrative indicators of alcohol-related harm, such as police incidents and hospitalisations, that potentially represent the more severe harms that are experienced by fewer community members and that are more susceptible to institutionalised racism in their reporting (e.g. evidence suggests that police incidents might be more readily coded as alcohol-related for Aboriginal people and that their experience of interpersonal discrimination contributes to their psychological distress) [28,29]. This may mean that increasing statistically significant reductions in alcohol-related harm using routinely collected measures will require community-action activities to be complemented by structural strategies that can be applied more comprehensively across whole populations, such as supply reduction strategies (e.g. earlier closing times for licensed venues or increasing the minimum legal age for purchasing or consuming alcohol), demand reduction strategies (e.g. greater restrictions on alcohol advertising or using taxes to increase the price of alcohol) and cultural safety training for a range of service providers [30,31]. Alternatively, it may reflect methodological issues, such as attempting to use routinely collected administrative data for purposes for which they are not intended (assessing program effects), or insufficient alignment between program components, the expected outcomes (as described in Fig. 2) and the measures of those outcomes.

Other methodological considerations may also have influenced the findings of this study. First, the order in which the communities commenced the program would ideally be randomised to control for possible order effects [12], although this can be difficult in participatory action studies where priority is given to community readiness and preferences. Second, the relatively brief funding period for this project meant that the community-based program needed to be developed, implemented and evaluated in three years, which limited the time for program co-design and implementation, and the number of data points available for analysis [32,33]. Third, the relatively small populations of communities 2 and 3 (1250 and 5700 respectively) meant that they had less outcome data available than is preferred for a robust interrupted time series analysis [32,33]. This suggests that more optimal application of interrupted time series analyses in smaller communities requires longer timeframes. Fourth, the variation in program effects reported across communities may be associated with different survey response rates (range 54 %–72 %), even though these response rates were higher than in a previous community-based alcohol study in rural NSW [21]. Alternatively, the variation in program effectiveness may reflect the different activities that each community selected to operationalise the three core program components (improving service engagement, promoting community activities and increasing community empowerment). Although the goal of this innovative program design is to optimise program effectiveness and generalisability (by defining programs using best-evidence core components that can be tailored to the circumstances of different settings), the specific impact of the combination of activities that operationalised the core components in each community was unmeasured (unlike fidelity, which assessed the extent to which activities were actually implemented in each community). Developing the capacity to measure the effectiveness with which the selected suite of community activities give effect to the core program components will be important in further refining this approach to the design of whole-of-community intervention programs led by communities.

Despite these limitations, the positive findings in this study most likely reflect the high level of collaboration between the researchers and the participating Aboriginal communities in every stage of the project [14]. This project demonstrated how the knowledge of communities and researchers can be combined to develop community-based programs that were successfully implemented, were grounded in the experiences of the participating communities and facilitated community control of the project budget. The acceptability of this approach to the communities is also evidenced by the sustainability of their selected activities. Midnight Basketball in community 1, for example, continued to operate for at least three years after the project ceased, Family Wellbeing groups are still provided on a weekly basis in community 1, and the holiday programme for at-risk youth in community 2 lead to the establishment of a permanent youth program.

Another strength of this project is that the program logic model (Fig. 2) is an especially innovative framework that allowed the program to be both standardised by core components and tailored to the specific characteristics of each participating community. The UK Medical Research Council recommends such a theoretical deconstruction of complex interventions [34] and this specific approach has now been successfully applied by the researchers in a range of settings, including Aboriginal residential rehabilitation services [35], community-based programs for high-risk young people [36] and community-based supported accommodation programs for men reintegrating into the community from prison [37].

Finally, it is noteworthy that these outcomes were not spread evenly across the three communities but were achieved more frequently in community 2 (n = 6 statistically significant improvements), compared to both community 1 (n = 2 statistically significant improvements) and community 3 (n = 1 statistically significant improvement). This may reflect greater levels of participation in community 2, or a preferential commencement date (the middle of the year), or that it was possible to introduce more targeted and effective program activities because it was the smallest and the most remote of the three communities, or that there was a stronger self-report bias associated with a smaller community.

## Author contributions

Conceptualisation: AS, BC and MS. Methodology: AS, BC and MS. Data curation or analysis: JO, BB, RB, CS, CM, SC and MS. Program design and implementation: MS, RB, BB and JO. Writing and editing: MS, AW, BC, AM and AS. Project administration: AS, MS, CS, BB, RB and JO. Funding acquisition: AS. All authors have read and agreed to the published version of the manuscript.

## Ethical statement

Ethics approval was granted by NSW Aboriginal Health & Medical Research Council's Ethics Committee (No: 987/13) and the NSW Population and Health Services Research Ethics Committee (No: 2014/02/516).

## Funding

This research was funded by the New South Wales Ministry of Health and author MS received a PhD scholarship from the National Drug and Alcohol Research Centre, UNSW Sydney.

## Declaration of competing interest

The authors declare that they have no known competing financial interests or personal relationships that could have appeared to influence the work reported in this paper.
